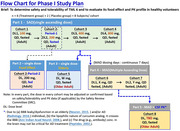# Design of A Phase 1 Trial for Safety, Tolerability, Single‐ascending Dose, Multiple‐ascending Dose, Food Effect, and Pharmacokinetic Study of TML‐6, A Novel Oral Multi‐Target Drug, in Healthy Volunteers

**DOI:** 10.1002/alz.084315

**Published:** 2025-01-09

**Authors:** Chien Hong Lin, Shang Hung Chen, Chia‐ Yu Hsu, Hui‐Chen Wang, Ih‐Jen Su

**Affiliations:** ^1^ Merry Life Biomedical Company, Ltd., Tainan City, Taiwan Taiwan; ^2^ National Health Research Institutes, Tainan City, Taiwan Taiwan; ^3^ Southern Taiwan University of Science and Technology, Tainan City, Taiwan Taiwan

## Abstract

**Background:**

Alzheimer’s disease (AD) is complex in pathogenesis and related to aging biology, especially in late‐onset AD. We identified a novel synthetic curcumin analog TML‐6 through the platform of 6 biomarkers of anti‐aging, anti‐inflammation, and anti‐Aβ as the potential AD drug candidate. TML‐6 exhibits multi‐target effects on AD pathogenesis, including the activation of NrF‐2, the regulation of autophagic machinery through mTOR, the inhibition of APP synthesis and reduction of Aβ, the upregulation of ApoE, and the inhibition of microglial activation. In the past years, we conducted series of preclinical studies on TML‐6 which showed satisfactory bioavailability and sufficient safety. US pre‐IND consultation has been completed, and IND/ phase I trial is expected to be approved on February, 2024.

**Method:**

Phase 1 trial was designed into 5 parts containing 8 cohorts on 72 healthy subjects, including single ascending doses and multiple ascending doses. The food effect, the elderly subjects, the plasma pK analysis, and the CSF pK analysis were studied.

**Result:**

The final phase I trial protocol was shown in the flow chart. The phase 1 trial will be conducted by CRO in Glendale Adventist Medical Center, LA, USA. According to pre‐clinical data, we anticipate good tolerability and safety of phase 1 trial through TML‐6 oral administration in healthy adults. The pK data will be analyzed for the design of dosing regimens in phase 2.

**Conclusion:**

TML‐6 exhibits a multi‐target action mechanism meeting the new era strategy to develop AD drug. The combined data of phase 1 trial and 9‐month chronic toxicology in dogs will be applied to US FDA for global phase 2a trial, with integration of blood biomarkers. We look forward to the success of TML‐6 in clinical trials that will provide an ideal therapy, with potential of combining with anti‐amyloid drug, for Alzheimer’s disease.

**Key Words**: TML‐6; curcumin analogue; Alzheimer’s disease; amyloid‐beta; aging biology